# Early stage breast cancer treatment and prognostic factors: Post San Antonio Breast Cancer Symposium 2016

**DOI:** 10.1007/s12254-017-0328-2

**Published:** 2017-04-25

**Authors:** Christoph Suppan, Marija Balic

**Affiliations:** Department of Oncology, Medical University of Graz, Auenbruggerplatz 15, 8036 Graz, Austria

**Keywords:** Early breast cancer, Endocrine therapy, CTC, HER2

## Abstract

Developments in early breast cancer presented at the 2016 San Antonio Breast Cancer Symposium included important studies on extending adjuvant endocrine therapy with aromatase inhibitors, the role of bisphosphonates, and evidence for molecular prognostic factors and circulating tumor cells in early stage breast cancer patients.

## Endocrine therapy

Adjuvant endocrine therapy is a milestone of treatment in hormone-receptor positive early breast cancer. Since more than 50% of disease relapses occur after the first 5 years of follow-up, the optimal duration of hormone therapy with an aromatase inhibitor is still unknown [1].

The DATA study, a multicenter phase III study, compared 6 years of anastrozole versus 3 years after 2–3 years of adjuvant tamoxifen in estrogen receptor/progesterone receptor (ER/PR)-positive breast cancer patients who were postmenopausal at randomization. In this study, 1912 patients were included. The primary endpoint of the study was adapted disease-free survival defined as the disease-free survival beyond the 3 years after randomization to therapy with an aromatase inhibitor (AI). The study was designed to detect an increase of the adapted disease-free survival (ADFS) in the group with extended AI therapy. The ADFS after 5 years was 79% in the 3‑year anastrozole treatment group and 83% in the group with 6 years of treatment with anastrozole, which was not statistically significant (*p = *0.07, HR 0.79%). In a subgroup analysis of ER-positive, PR-positive, HER2-negative, pN+ patients who received adjuvant chemotherapy, a significant difference in the 5‑year ADFS could be shown (5-year DFS 86 vs. 75.9%, hazard ratio [HR] 0.58, *p = *0.01). Therefore, this selected group of patients might benefit from an extended use of adjuvant AI after 5 years of sequential endocrine therapy, whereas the overall findings do not support this strategy for all postmenopausal hormone receptor-positive breast cancer patients [2].

In the IDEAL trial, patients were randomized to either 2.5 or 5 years of letrozole therapy after initial 5 years of tamoxifen and/or AI. The study included 1824 patients. The disease-free survival (DFS) and overall survival (OS) in the group of patients with an extended adjuvant AI therapy of 5 years was not significantly better than in the group with AI extension of 2.5 years, and therefore, this study showed no benefit of AI-based adjuvant therapy longer than a maximum of 7.5 years. Despite this aspect, the IDEAL trial showed a significant difference in prevention of second primary breast cancer (HR 0.37, absolute risk reduction of 1% at 5 years) [3].

NSABP B‑42 compared 5 years of letrozole versus placebo after 5 years of AI or tamoxifen followed by AI in 3966 women. DFS in the letrozole arm (84.7%) was not significantly better than in the placebo arm (81.3%) with a hazard ratio of 0.85 (*p = *0.048). However, extended letrozole therapy resulted in a nonsignificant reduction of 15% in the risk of a disease-free survival event and the study demonstrated a statistically significant reduction of 29% in breast cancer-free interval (BCFI) events and a statistically significant 28% decrease in distant recurrence (DR) with extended letrozole therapy compared with placebo (HR for BCFI, 0.71; 95% CI 0.56–0.90; *p = *0.003 and HR for DR, 0.72; 95% CI, 0.56–0.90; *p = *0.03). Comparing to the results from other trials such as DATA or IDEAL and a benefit in selected patients with tumor characteristics such as node positivity, it should be mentioned that 57–58% of patients in NSABP B‑42 were node negative [4].

In the discussion of the three trials of prolonged AI therapy, Prof. Dr. Michael Gnant suggested that despite the negative primary end point analyses of the trials some interesting signals about potential counselling strategies for individual patients can be derived. The decision in favor of prolonged therapy should be based on a careful consideration depending on bone health, younger age, and higher risk by clinicopathological factors such as node-positive tumors with a more luminal type.

## Adjuvant bisphosphonates

Bisphosphonates have been studied in randomized trials in early breast cancer to investigate their ability to prevent cancer treatment-induced bone loss and reduce the risk of disease recurrence and metastasis. Several studies so far have suggested a benefit from the addition of bisphosphonates in the adjuvant setting, in particular in the population of real postmenopausal patients [5]. Although these treatment benefits have been reported, bisphosphonates do not currently have regulatory approval for these indications. European experts recommend the addition of bisphosphonates (either intravenous zoledronic acid or oral clodronate) to be considered as a part of adjuvant breast cancer treatment in this population, providing that potential benefits and risks are discussed with patients [6]. At SABCS this year, another trial on adjuvant bisphosphonates was presented by Dr. Vliek. In the trial TEAM IIb 1116 postmenopausal women with stage I–III breast cancer and adjuvant hormone therapy were included and randomized to treatment with or without ibandronate in addition to hormonal treatment for 3 years. There was an increase of DFS at 3 years from 90.8 to 94.3% which was not statistically significant. One limitation of the study was that the study had to be amended due to a slow accrual. Although the magnitude of benefit was comparable to previous positive studies, the study is considered negative since it did not reach the predefined endpoint. Furthermore, at the time point of analysis, there are still 73 patients receiving ibandronate [7].

## HER2-positive BC

The NSABP B‑52 trial evaluated pathologic complete response rates in patients with hormone receptor and HER2-positive breast cancer treated with neoadjuvant therapy of docetaxel, carboplatin, trastuzumab, and pertuzumab (TCHP) with or without concurrent estrogen deprivation therapy. The hypothesis of the study was that concurrent inhibition of ER and HER2 plus chemotherapy would not be antagonistic but it would overcome resistance to treatment and improve pCR rates.

The pathologic complete response (pCR) rate in the breast and nodes as the primary endpoint of the study was 40.9% in the group with TCHP alone and 46.1% in the group with concurrent estrogen deprivation (*p = *0.36). Despite the fact that there was no increased toxicity in the combination arm, the numerically improved pCR rates could not reach statistical significance [8]. Further evaluation may add to the knowledge whether it will be possible to define a subgroup of patients likely to derive benefit from such approach, while on the other hand some selected patients could be treated with a de-escalating approach.

Several studies on the optimal treatment of HER2-positive early breast cancer were discussed. The APT trial enrolled patients with HER2-positive, ER-positive, or negative node negative breast cancer with a size <3 cm receiving weekly paclitaxel and weekly trastuzumab (2 mg/kg) twelve times, followed by thirteen 3‑weekly doses of trastuzumab. This therapy regime turned out to be a reasonable and appealing approach for the majority of patients with stage I HER2-positive breast cancer showing a 3-year DFS of 98.7% [9]. The results of the Aphinity trial with adjuvant pertuzumab are awaited and, therefore, both the regimens ACTH (anthracycline/cyclophosphamide/taxane/trastuzumab) and TCH (docetaxel/cyclophosphamide/trastuzumab) remain the standard of care in stage II/III HER2-positive breast cancer.

Furthermore, an update on the I‑SPY2 trial, with focus on preoperative weekly paclitaxel plus or minus addition of targeted therapies, followed by doxorubicin and cyclophosphamide was presented. I‑SPY 2, the Investigation of Serial Studies to Predict Your Therapeutic Response through Imaging and Molecular Analysis 2, is a platform trial that compares multiple experimental groups to a standard neoadjuvant chemotherapy backbone in high-risk breast cancer subtypes. In contrast to an open-label phase II trial, there is no fix statistical assumption that determines the sample size. Biomarker assessment (based on HER2 receptor and hormone receptor expression status as well as the 70-gene assay) at baseline classifies patients into predefined groups [10]. For triple-negative breast cancer in this trial, addition of veliparib and carboplatin substantially increased the pathologic complete response (pCR) rate, although there was concern about an imbalance of BRCA+ in the two arms. Three gene expression signatures, PARPi-7, BRCAness and MP 1/2 class were shown as predictors of response to veliparib and carboplatin, demonstrating that in the future we may look at selected genomic signatures and not only rely on ER, PR, and HER2 [11].

In the adjuvant setting of HER2-positive breast tumors, results of KAITLIN, a phase-3-trial comparing T‑DM1 and pertuzumab for 1 year after AC or FEC to trastuzumab and pertuzumab after receiving AC or FEC and twelve weekly-doses of taxane are awaited. So far, the current standard in the adjuvant systemic treatment of early HER2-positive breast cancer still consists of chemotherapy plus 12 months of trastuzumab, with or without endocrine therapy. Extending the treatment with 1 year of neratinib, a pan-HER inhibitor, was demonstrated recently in the ExteNET trial, but this study has not yet had an impact on clinical practice. Preliminary follow-up data suggest improvement in disease-free survival with higher toxicity and an unclear persisting benefit after pertuzumab. The outcome of the APHINITY trial evaluating the addition of pertuzumab to trastuzumab for 12 months postoperatively should be presented in the next few months, potentially changing the current practice stated above.

## Risks of invasive cancer and metastasis

### DCIS

There is an ongoing controversy regarding the risk of development of invasive breast cancer from ductal carcinoma in situ (DCIS) and the needed treatment in order to minimize this risk and ultimately the risk of death. Multidisciplinary consensus panel considered optimal surgery and negative margin width of 2 mm for DCIS treated with breast-conserving surgery and whole-breast irradiation as the standard for an adequate surgery, as it is associated with lower rates of ipsilateral breast tumor recurrence. However, in cases of not achieving a 2-mm margin other factors, such as residual calcifications on mammography, the extent of DCIS on the margin, and age expectancy, should be considered [12]. One study validated a biological risk signature-based test to assess ipsilateral breast event risk after breast-conserving surgery with or without radiation therapy. Patients were stratified into clinically relevant low and elevated risk groups (≤3 vs. >3) in an independent validation of a Prelude DCIS test. Patients in the elevated risk group had a higher likelihood of 10-year total ipsilateral breast event. Although the number of patients treated with breast-conserving surgery without radiotherapy was limited and while the stratification by risk group for those without radiotherapy was in the expected direction, it did not reach statistical significance [13].

## Prognostic factors

Since there is a substantial number of overtreated patients with early breast cancer in the adjuvant setting, tools such as Adjuvant! Online or PREDICT plus have been developed in order to assist in therapy decision making which is normally based on characteristics of the tumor and the patient [14, 15]. The MINDACT trial, published in the New England Journal of Medicine in August 2016, is the first prospective clinical trial investigating the clinical utility of a genomic test for clinical decision making. All patients were categorized in “low genomic risk” or “high genomic risk” by MammaPrint, a commercially available genomic test that assesses the risk for recurrence. Furthermore, patients were divided into “low clinical risk” and “high clinical risk” according the 10-year probability of breast cancer-specific survival (10-year BCSS) without systemic therapy defined by a modified version of Adjuvant!Online. At SABCS this year, the results of a preplanned MINDACT substudy, which compared the outcome based on molecular subtyping to surrogate pathological subtyping, were presented [16]. The speaker concluded that molecular subtyping seemed to be better correlated with outcome than pathological classification, though limited by low numbers of patients in each subgroup. In the subgroup of luminal patients, the number of patients with the luminal A subtype determined by genomic profiling was numerically larger. As demonstrated in the supplements of the manuscript [17], the estimated 5‑year distant metastasis-free survival for the c‑high/g-low group without adjuvant chemotherapy was 94.7% (95% CI = 92.5–96.2%). In the original publication of the MINDACT trial, it was successfully demonstrated that in this subpopulation the chemotherapy may be safely omitted. Moreover, centrally assessed Ki67 labeling index of 20% may be the best cut-off for surrogate differentiation between luminal A and B.

## Circulating tumor cells

In the field of circulating tumor cells (CTC), an international meta-analysis of CTC detection in early breast cancer patients treated by neoadjuvant chemotherapy (IMENEO study) was presented. Data of 2156 individual patients from 21 studies and 16 centers worldwide were collected. The primary endpoint was overall survival; secondary endpoints included distant disease-free survival (DDFS), locoregional relapse-free interval (LRFI), and pathological complete response (pCR). Nonoverlapping CTC time points were the following: baseline (5–0 weeks before neoadjuvant chemotherapy), 1–8 weeks after start of neoadjuvant chemotherapy, 5–0 weeks before surgery, and 1–52 weeks after surgery. In univariate analyses, ≥1 CTC at baseline was a prognostic factor for overall survival (HR = 2.6 [1.9–3.4], *p < *0.0001), distant disease-free survival (HR = 2.4 [1.9–3.1], *p < *0.0001), and for locoregional relapse-free interval (HR = 1.8 [1.2–2.7], *p = *0.001). In multivariate analyses, baseline CTC detection was an independent prognostic factor for OS, DDFS, and LRFI, together with pCR, cT, cN, and tumor subtype, which shows that CTC are a prognostic biomarker in early breast cancer treated by neoadjuvant chemotherapy with a high level of evidence [18].

## Conclusion

In the field of adjuvant endocrine therapy with an aromatase inhibitor, an individual procedure for every patient in case of extending treatment over 5 years is important. Trastuzumab for 12 months is still standard of care in adjuvant treatment of HER2-positive tumors.

## References

[1] Pan H, Gray RG, Davies C (2016). Predictors of recurrence during years 5–14 in 46,138 women with ER+ breast cancer allocated 5 years only of endocrine therapy (ET). J Clin Oncol.

[2] Tjan-Heijnen VC, Van Hellemond IE, Peer PG (2016). First results from the multicenter phase III DATA study comparing 3 versus 6 years of anastrozole after 2–3 years of tamoxifen in postmenopausal women with hormone receptor-positive early breast cancer.

[3] Blok EJ, van de Velde CJH, Meershoek-Klein Kranenbarg EM (2016). Optimal duration of extended letrozole treatment after 5 years of adjuvant endocrine therapy; results of the randomized phase III IDEAL trial (BOOG 2006–05).

[4] Mamounas EP, Bandos H, Lembersky BC. et al. A randomized, double-blinded, placebo-controlled clinical trial of extended adjuvant endocrine therapy (tx) with letrozole (L) in postmenopausal women with hormone-receptor (+) breast cancer (BC) who have completed previous adjuvant tx with an aromatase inhibitor (AI): Results from NRG Oncology/NSABP B‑42. Abstract, Breast Cancer Symposium, San Antonio. 2016, pp 1–5.

[5] Coleman R, Gray R, Powles T (2015). Adjuvant bisphosphonate treatment in early breast cancer: meta-analyses of individual patient data from randomised trials. Lancet.

[6] Hadji P, Coleman RE, Wilson C (2016). Adjuvant bisphosphonates in early breast cancer: consensus guidance for clinical practice from a European Panel. Ann Oncol.

[7] Vliek SB, Meershoek-Klein Kranenbarg E, van Rossum AGJ (2016). The efficacy and safety of the addition of ibandronate to adjuvant hormonal therapy in postmenopausal women with hormone-receptor positive early breast cancer. First results of the TEAM IIB trial (BOOG 2006–04).

[8] Rimawi MF, Reena S, Checchini M (2016). A phase III trial evaluating pCR in patients with HR+, HER2-positive breast cancer treated with neoadjuvant docetaxel, carboplatin, trastuzumab, and pertuzumab (TCHP) ± estrogen deprivation: NRG oncology/NSABP B‑52. General session 3.

[9] Tolaney SM, Barry WT, Dang CT (2015). Adjuvant paclitaxel and trastuzumab for node-negative, HER2-positive breast cancer. N Engl J Med.

[10] Bartsch R, de Azambuja E (2016). I-SPY 2: optimising cancer drug development in the 21st century. ESMO Open.

[11] Wolf DM, Yau C, Sanil A (2016). DNA repair deficiency biomarkers and MammaPrint high1/(ultra)high2 risk as predictors of veliparib/carboplatin response: results from the neoadjuvant I‑SPY 2 trial for high risk breast cancer.

[12] Morrow M, Van Zee KJ, Solin LJ (2016). Society of Surgical Oncology-American Society for Radiation Oncology-American Society of Clinical Oncology Consensus guideline on margins for breast-conserving surgery with whole-breast irradiation in ductal carcinoma in situ. Pract Radiat Oncol.

[13] Bremer T, Whitworth P, Leo M (2016). DCIS biological risk profile predicts risk of recurrence after breast conserving surgery in a Kaiser Permanente NW population.

[14] Olivotto IA, Bajdik CD, Ravdin PM (2005). Population-based validation of the prognostic model ADJUVANT! for early breast cancer. J Clin Oncol.

[15] Wishart GC, Bajdik CD, Dicks E, Provenzano E (2012). PREDICT Plus: development and validation of a prognostic model for early breast cancer that includes HER2. Br J Cancer.

[16] Cardoso F, Slaets L, de Snoo F (2016). Can surrogate pathological subtyping replace molecular subtyping? Outcome results from the MINDACT trial.

[17] Cardoso F, van’t Veer LJ, Bogaerts J, Slaets L (2016). 70-gene signature as an aid to treatment decisions in early-stage breast cancer. N Engl J Med.

[18] Bidard F-C, Michiels S, Mueller V (2016). IMENEO: International MEta-analysis of circulating tumor cell detection in early breast cancer patients treated by neoadjuvant chemotherapy.

